# Ligament Tissue Engineering and Its Potential Role in Anterior Cruciate Ligament Reconstruction

**DOI:** 10.1155/2012/438125

**Published:** 2011-12-29

**Authors:** E. W. Yates, A. Rupani, G. T. Foley, W. S. Khan, S. Cartmell, S. J. Anand

**Affiliations:** ^1^Department of Trauma and Orthopaedics, Stepping Hill Hospital, Stockport NHS Foundation Trust, Stockport, SK2 7JE, UK; ^2^Materials Science Centre, University of Manchester, Oxford Road, Manchester, M13 9PT, UK; ^3^Royal National Orthopaedic Hospital and Institute of Orthopaedics and Musculoskeletal Science, University College London, Stanmore, Middlesex, HA7 4LP, UK

## Abstract

Tissue engineering is an emerging discipline that combines the principle of science and engineering. It offers an unlimited source of natural tissue substitutes and by using appropriate cells, biomimetic scaffolds, and advanced bioreactors, it is possible that tissue engineering could be implemented in the repair and regeneration of tissue such as bone, cartilage, tendon, and ligament. Whilst repair and regeneration of ligament tissue has been demonstrated in animal studies, further research is needed to improve the biomechanical properties of the engineered ligament if it is to play an important part in the future of human ligament reconstruction surgery. We evaluate the current literature on ligament tissue engineering and its role in anterior cruciate ligament reconstruction.

## 1. Introduction

Ligamentous tissue is composed of fibroblasts and an extracellular matrix. Whilst the fibroblast is the main cell type, the extracellular matrix is predominantly types I and III collagen with proteoglycans, water, and small amounts of elastin present [1, 2]. The fibroblasts secrete the extracellular matrix and also maintain, repair, and regenerate new tissue growth. Whilst there are no specific markers to differentiate ligament from tendon, the total amount of collagen, elastin, and proteoglycans varies between the two tissues as well as the types of collagen. The process of ligament healing is complex and growth factors play an important role [2]. Most studies concentrate on the anterior cruciate ligament (ACL) repair as it is one of the most common injuries and is therefore a good model for illustrating ligament repair or regeneration [3–7]. The ACL plays an essential role in the smooth motion and stability of the knee joint and, due to its poor vascularity, has limited healing capacity [4, 5]. Traditionally, ACL ruptures have been treated using autografts, allografts, and synthetic grafts made from polymers. All of these techniques have a number of disadvantages [4–8].

The speciality of orthopaedics lends itself to tissue engineering. Musculoskeletal tissues are often injured or lost in trauma and disease and demonstrate limited healing potential. Whilst orthopaedic surgery has advanced in the use of cartilage replacement, it remains to be seen whether there will be a shift from tissue replacement towards tissue regeneration [9]. Tissue engineering offers an unlimited source of natural tissue substitutes. By using appropriate cells, biomimetic scaffolds, and advanced bioreactors, it is possible that tissue engineering could be implemented in the repair and regeneration of tissue such as bone, cartilage, tendon, and ligament [10]. The use of cells significantly improves the construct quality, and in vivo injections of cells into the injured ligament can accelerate the repair process by laying down extracellular matrix, releasing growth factors, and triggering the necessary immune responses [4, 11]. Novel approaches are being tried including stem cell therapies, use of growth factors, mechanical loading, and gene therapy to achieve this end point [1, 2, 11–14].

## 2. Cell Source and Different Approaches in Ligament Tissue Engineering

Whilst it is imperative to use an appropriate cell type to achieve a functional ligament construct, little is known about the optimal cell source for ligament tissue engineering [4]. Ligament cells from different sources vary in their growth, dexamethasone responsiveness, and cell surface marker expression. All of these factors are important in enabling the tissue engineers to carefully select the optimal cell source and hence maximise efficacy [15]. The actual source of the cell, the variation in the behaviour of cells from different species, the passage number, and the animal model experiments must also be considered [5, 16]. The options available are mesenchymal stem cells (MSC) or primary fibroblasts derived from ligaments such as the ACL or medial collateral ligament (MCL) [4, 16–19].

Stem cells are immature biological cells which have the ability to proliferate, differentiate, and regenerate tissues. The two main types of stem cells in mammals are embryonic stem cells (ESC), formed a matter of days after egg fertilisation, and nonembryonic stem cells (non-ESC). Nonembryonic stem cells are also referred to as adult stem cells and are usually obtained from the bone marrow of adults. There are two types of stem cells available from this source: haemopoietic, which differentiate into blood cells, and MSCs. The less mature sources of MSCs such as the placenta and the umbilical cord blood are still considered non-ESCs whilst fetal stem cells are considered an intermediate cell type [20, 21].

Since the discovery of MSCs in 1976, it has become more apparent that their capacity to repair tissue is due to their ability to secrete soluble factors which alter the tissue microenvironment. A number of chemokines and cytokine receptors have been implicated in guiding the MSCs to the zone of tissue injury to allow tissue repair to begin. Despite this, there is little evidence with regard to the mechanism of mobilisation of MSCs from the bone marrow. One of their most important features is their unexplained immunological properties. Adult MSCs express moderate levels of class I major histocompatibility complex (MHC) proteins but no class II proteins. Their nonimmunogenicity indicates that immunosuppressive therapy is not required if transplanted into an allogenic host [20].

Bone marrow is the most popular source used to acquire MCSs due to its relative ease of access. The MSCs of bone marrow have a greater transdifferentiation capability compared to the MSCs of different tissue origin. However, bone marrow aspiration is invasive, potentially painful, and associated with increased risks of morbidity and infection. Other sources for MSCs have been discovered including amniotic fluid, umbilical cord blood, adipose tissue, synovium, and tendon but it is currently unclear which lineages these MSCs can differentiate into [20, 22].

## 3. Mesenchymal Stem Cells in Ligament Tissue Engineering

The literature would suggest that MSCs are the preferred method of ligament tissue engineering because they can easily differentiate into ligament fibroblasts after two weeks [1, 2, 4, 5, 12, 14, 16, 18, 23–26]. In large animal model experiments involving pigs, MSCs (passage 2) exhibited fibroblast phenotype and differentiation at 24 weeks postoperatively with silk-based scaffolds [5]. Most of the studies use MSCs at passage 2 [5, 12, 27, 28]. Following passages 2 and 3 at 25–30 days, rabbit MSCs have been shown to have stopped proliferation, increased in size, and assumed an irregular morphology. However, this might not be true for hMSCs [4]. Interestingly, Ge et al. found no difference in collagen production between passage 1 and 2 using rabbit MSCs [4]. Furthermore, it has been reported that MSCs lose their potential for osteogenic differentiation after passage 5-6 due to senescence or fibroblast contamination [29]. This may have important implications in ligament tissue engineering where MSCs are expected to differentiate into fibroblastic lineage and therefore achieve pure MSC culture. There are currently no specific markers that can reliably distinguish between MSCs and fibroblasts [29].

There is also the issue regarding the source of MSCs. Traditionally, MSCs have been harvested from bone marrow and other sources such as adipose tissue and cord blood. The potential role for harvesting MSCs from synovial fluid in ligament regeneration has also been reported [25, 30]. The number of MSCs is known to increase following any ligament injury and in degenerative disorders such as osteoarthritis [30, 31]. Cheng et al. reported better outcomes from the stem cells derived from the ACL itself compared to bone-marrow-derived MSCs [32].

A bioreactor uses various combinations of chemical, mechanical, electrical, or magnetic stimulation to accelerate the process of differentiation of MSCs into the fibroblastic lineage and facilitate the development of a de novo tissue construct that is comparable to the desired tissue [33].

There are number of key bioreactor principles that must be obeyed for the bioreactor to function successfully. The bioreactor should be designed to operate under strict sterile conditions in order to prevent contamination of the neotissue with microorganisms. The bioreactor should maintain accurate control of the physiological environment of the tissue culture. This ensures control of parameters such as pH values, oxygen concentrations, temperature and metabolite concentrations. The bioreactor should provide the culture with the fundamental nutrients and gases. Finally, the bioreactor should be able to accommodate the culture of more than two cell types at the same time. This is particularly relevant when engineering complex tissues [33].

Chemical stimulation techniques employ a cocktail of polypeptides known as growth factors. Growth factors such as transforming growth factor-*β* (TGF-*β*) and epidermal growth factor (EGF), TGF-*β* and insulin (maybe in a sequential approach), insulin-like growth factor (IGF-I), basic fibroblast growth factor (bFGF), vascular endothelial growth factor (VEGF), platelet-derived growth factor (PDGF), or growth and differentiation factor (GDF) can expedite the MSCs to differentiate into fibroblasts and also improve the cell proliferation and extracellular matrix deposition [1, 12, 25, 27, 34]. Sustaining sufficient quantities of growth factor within the local tissue has been difficult until the introduction of gene transfer technology [21]. Wei et al. experimented by transfecting bone-marrow-derived MSCs with adenovirus vector encoding TGF-*β*1, VEGF, or TGF-*β*1/VEGF before surgical implantation into experimental ACL grafts [24]. They found that this combination significantly improved the performance of the MSCs by promoting angiogenesis. The best mechanical properties were achieved at 24 weeks.

Mechanical conditioning is another method used to induce differentiation of MSCs into the fibroblast lineage. Triggering the cell surface stretch receptors results in activation of the intracellular signalling cascades leading to synthesis of the necessary extracellular matrix proteins [12, 27, 28]. Altman et al. developed a specialised bioreactor for this purpose and found that helically organised collagen fibres formed in the direction of the load [28]. Cocultures are rapidly becoming popular to promote MSC differentiation by growing them together with fibroblasts [12, 35, 36]. The mechanism of action is based both on the cell-to-cell interactions between the fibroblasts and MSCs and on the cytokines released within the 3-dimensional environment. The differentiated MSCs are also stimulated to secrete more extracellular matrix [12]. In one study, fascia was wrapped around the MSC-seeded ACL tissue construct and, whilst this promoted extracellular matrix production, it did not enhance the ultimate tensile load and stiffness [37].

Experiments using electromagnetic stimulation techniques have been carried out and demonstrate positive findings. In one study, a single shot of low-energy laser therapy was administered to the medial collateral ligament of a rat resulting in a significant increase in the collagen fibril size. Another study reported an increase in osteoblastic and alkaline phosphatase activity when electrical stimulation was applied to rabbit bone marrow [33].

Oe et al. studied ligament regeneration in rats following intra-articular injection of either fresh bone marrow cells (BMCs) or cultured MSCs 1 week after partial ACL transection. At 4 weeks, donor cells were detected within the transected ACLs in both the BMC and MSC groups and the ACLs exhibited almost normal histology. Furthermore, there were significantly more mature spindle cells, near normal biomechanical properties and higher levels of TGF-*β* in the ACL tissue of the BMC group. They concluded that direct intra-articular bone marrow transplantation is an effective treatment for partial ruptures of the ACL [3]. Similar results using intra-articular injections have been reported by other researchers [38]. Lim et al. performed ACL reconstructions in adult rabbits using hamstring tendon autografts which were coated with MSCs in a fibrin glue carrier. At 8 weeks, good osteointegration was observed and they performed significantly better on biomechanical testing than the controls [39].

In summary, MSCs of low passage number are a good source of cells for use in ligament regeneration. The advantages include: the use of autologous cells, the relative ease of procurement and growth in the lab and the ability to differentiate into fibroblasts at around 2–4 weeks and secrete the extracellular matrix.

## 4. Primary Fibroblasts in Ligament Tissue Engineering

Fibroblasts are another choice of cell and can be harvested from different sources. Cooper et al. concluded that ACL-derived fibroblasts were the most suitable cells for the further study and development of tissue-engineered ligament as opposed to the cells derived from the MCL, achilles tendon, or patellar tendon [40]. Another study compared the performance of fibroblasts extracted from both intact and ruptured human ACLs. They observed that cells extracted from the ruptured ACL were more useful in ligament tissue engineering [17]. Fibroblasts from other sources such as the skin are also being tested for their use in ligament tissue engineering. However, there is debate as to how well they are able to function given the change from their normal physiological environment [4, 41].

## 5. Comparison between Mesenchymal Stem Cells and Primary Fibroblasts

Ge et al. compared the performance of fibroblasts isolated from the ACL and MCL to that of bone-marrow-derived MSCs in rabbits. He found that the proliferation rate and collagen production were higher with MSCs (passage 1–37.1 mg/mL and passage 2–36.4 mg/mL) than with fibroblasts (ACL 23.2 mg/mL and MCL 19.8 mg/mL). The cells survived at least 6 weeks in the knee joint and in the MSC group, survivorship was due to the protection of the surrounding fascial covering which led to a mild immune response. All three groups expressed equal amounts of collagen I, collagen III, and *α*-smooth muscle actin [4]. Liu et al. also found that MSCs grew faster than fibroblasts on silk scaffolds. Furthermore, the gene expression for transcripts and production was increased in the MSC group compared to the fibroblast group [16].

## 6. Response of Cells to Different Biomaterials in Ligament Tissue Engineering

Stem cells are commonly seeded or implanted into a construct that is capable of providing structural support to three-dimensional tissue growth. These constructs or scaffolds facilitate tissue formation by enabling cell migration, proliferation, and differentiation [9, 22].

The ideal scaffold should have some key properties. The scaffold should be able to bridge any complex three-dimensional anatomical defect, and this can be achieved using surgical experience or through sophisticated computer mapping systems. The scaffold must provide temporary mechanical support until the three-dimensional neotissue has regenerated to a mature enough state that the tissue is able to bear load. A scaffold that is biodegradable is often desirable because absorption by the surrounding tissue prevents the need for surgical removal. However, the rate of absorption must mirror the rate of neotissue formation. This allows the scaffold to provide a temporary structural mechanical support until the newly formed tissue takes over the mechanical load. Porous scaffolds enhance tissue regeneration by delivering biofactors. Pore diameter is important in facilitating cell migration, proliferation, and growth factor movement. It is important to get the right balance between tissue regeneration and the mechanical properties of the scaffold. Whilst smaller pores are inefficient, larger pores can compromise the mechanical properties of the scaffold [21, 22].

Scaffolds can be composed from naturally occurring material or from synthetic material. Studies have evaluated the ability of different components within the extracellular matrix to support cell growth. Proteinaceous material such as collagen and polysaccharidic material such as glycosaminoglycans (GAGs) have been found to be suitable with regard to cell compatibility, but immunogenicity remains a potential problem. Polylactic acid (PLA) is a commonly used synthetic scaffold which easily degrades within the human body by forming lactic acid. Materials such as polycaprolactone (PCL) and polyglycolic acid (PGA) degrade in a similar way to PLA but exhibit different rates of degradation. Research continues in the quest to create a scaffold that combines the advantages of both groups of biomaterials [21, 22].

Scaffolds that are used in ligament tissue engineering should mimic the extracellular matrix both by providing appropriate mechanical support and by promoting cellular adhesion and proliferation [5, 25]. The biomaterials commonly used are silk, a variety of polymers (especially polyhydroxyesters), and natural substrates such as collagen, gelatine, small intestinal submucosal extracellular matrix, and even decellularised ligaments [5–7, 17, 23, 25, 34, 42, 43]. In a study by Ouyang et al., the adhesion, proliferation, and morphology of rabbit ACL cells and MSCs were investigated using different biodegradable polymeric films. They found that high molecular weight poly (dl-lactide-co-glycolide) was most favourable for cell attachment and proliferation and that MSCs performed better than ACL cells [44]. Knitting, braiding, and electrospinning are all popular techniques used in the manufacture of biomimetic fibrous scaffolds for ligament tissue engineering [5, 6, 19, 23, 42, 43]. The cells are proven to spontaneously orientate along the direction of the fibres leading to abundant extracellular matrix secretion rich in collagens I and III [5, 42].

Silk is becoming increasingly popular due to its good biocompatibility, slow degradability, and excellent mechanical properties [5, 6, 34, 45]. Altman et al. popularised silk scaffolds for ligament tissue engineering [45]. They showed a significant increase in the number of cells and matrix production after culturing human MSCs for 14 days. Furthermore, mRNA analysis demonstrated a similar gene expression to native ligament cells. Composite scaffolds comprising silk are extremely biocompatible and both MSCs and primary fibroblasts can attach to them within 18 hours and proliferate profusely to secrete extracellular matrix [5, 16, 23, 25, 46]. Sahoo et al. developed a composite electrospun nanofibrous PLGA on knitted microfibrous silk scaffolds. They were coated with bioactive bFGF, the controlled release of which was dependent on the degradation of the fibres, facilitating MSC attachment, cell proliferation, and fibroblastic differentiation. This was proven by the upregulation of ligament specific extracellular matrix proteins by 14 days [25, 26]. bFGF is known to stimulate MSC proliferation and differentiation by acting synergistically with the mechanical stimulation and nanotopographic cues of the scaffold [25].

## 7. Methods of Characterising the Tissue Engineered Ligament

Cellular proliferation, protein synthesis, and extracellular matrix production are important aspects of ligament tissue engineering. Characterisation can be performed by histotechniques, quantifying the extracellular matrix protein (perhaps by immunostaining), scanning electron microscopy, or quantitative polymerase chain reaction for gene expression of relevant ligament-related proteins such as collagen type I and relevant transcription factors such as scleraxis, tenomodulin, and tenascin-C [5, 14, 16, 23, 27, 34, 42, 46–48]. In the case of MSCs, the protein transcription levels increase by 2 weeks [16]. Stress-strain tensile testing can be performed for tissue engineered ligaments to assess their mechanical efficiency [42].

## 8. Conclusion

Stem cells, growth factors, mechanical loading, biomimetic scaffolds, and gene therapy all play important roles in the quest to engineer the ideal ligament neotissue. Whilst repair and regeneration of ligament tissue has been demonstrated in animal studies, further research is needed to improve the biomechanical properties of the engineered ligament if it is to play an important part in the future of human ligament reconstruction surgery.

Ultimately, randomised controlled trials on human populations will be required to demonstrate the clinical application of the engineered ligament. Furthermore, a cost-benefit analysis will be necessary to justify its use over conventional ACL reconstruction surgery.
